# Empathetic responses by AI legal assistants and user over-disclosure: evidence from an online survey experiment

**DOI:** 10.3389/fpsyg.2026.1964996

**Published:** 2026-10-13

**Authors:** Siyuan Wang

**Affiliations:** School of Humanities, Jiyang College of Zhejiang A&F University, Zhuji, Zhejiang, China

**Keywords:** AI legal assistant, coded non-essential disclosure score, empathetic response style, online legal consultation, over-disclosure intention, parasocial response, privacy boundary judgment, socioemotional cues

## Abstract

**Introduction:**

Generative AI legal assistants rely on users to describe events, evidence, and transaction histories, yet the same accounts may contain private information that is unnecessary for legal guidance. Empathetic language may affect how selectively users decide what to provide.

**Methods:**

A one-factor between-subjects online survey experiment on Wenjuanxing randomly assigned 360 participants to rational, mildly empathetic, or highly empathetic AI legal responses (n = 120 per condition). Participants completed 1-5 Likert-type measures of parasocial response, privacy boundary judgment, and over-disclosure intention, together with related relational measures, and then answered an open-ended disclosure task.

**Results:**

The empathy manipulation was successful, F_(2,357)_ = 524.170, *p* < 0.001, and η^2^ = 0.746. Stronger empathetic responses increased parasocial response (η^2^ = 0.428), reduced privacy boundary judgment (η^2^ = 0.242), and increased over-disclosure intention (η^2^ = 0.406). The coded non-essential disclosure score rose from 0.994 in the rational condition to 6.132 in the highly empathetic condition, F_(2,357)_ = 170.795, *p* < 0.001, and η^2^ = 0.489. Necessary case facts remained stable, F_(2,357)_ = 0.245, *p* = 0.783. Bootstrap analysis yielded an indirect effect through parasocial response of 0.214, 95% CI [0.151, 0.283], and a sequential indirect effect through parasocial response and privacy boundary judgment of 0.063, 95% CI [0.033, 0.098].

**Discussion:**

Within this consumer-dispute scenario, stronger empathetic cues reduced users' selectivity about which information was appropriate to provide. Data-minimization prompts and evidence-oriented questioning may help retain limited emotional support without encouraging unnecessary disclosure.

## 1 Introduction

People increasingly turn to generative AI legal assistants for help with consumer transactions, wage claims, rental deposits, platform complaints, and contract disputes. These systems explain rules, organize evidence, suggest communication strategies, and outline procedural steps for users who may not initially consult a lawyer (Surden, 2019; Remus and Levy, 2017; Katz et al., 2024; Dahl et al., 2024; Magesh et al., 2025). Each task depends on the user supplying enough factual detail for the assistant to identify the dispute and respond to it.

Dates, transaction amounts, communication records, platform decisions, and the other party's conduct can determine how a legal rule applies and which evidence is useful. Users often embed those facts in broader narratives that include identity details, financial circumstances, family or work relationships, third-party information, account records, and psychological pressure (Nissenbaum, 2004; Petronio, 2002; Acquisti et al., 2015; Solove, 2024). Details that do not advance the legal task increase privacy exposure on the input side of legal AI, alongside the better-known risks created by inaccurate output (Dahl et al., 2024; Magesh et al., 2025).

Expressions of understanding, reassurance, patience, and companionship can make users more willing to continue an exchange. Research on relational agents and service chatbots links these cues to perceived understanding, engagement, trust, and self-disclosure (Bickmore and Picard, 2005; Araujo, 2018; Adam et al., 2021; Ho et al., 2018; Zhang et al., 2024). During legal consultation, empathetic language may also influence whether users regard private background and information about other people as appropriate parts of the account.

Parasocial interaction and social response research describe how users can experience relational closeness with a mediated system during a specific exchange (Horton and Wohl, 1956; Reeves and Nass, 1996; Nass and Moon, 2000). Human–AI trust research separates that closeness from confidence in an assistant's competence and benevolence (McKnight et al., 2002; Lee and See, 2004; Glikson and Woolley, 2020). Privacy boundary research examines how recipients, purposes, information types, and expected information flows enter disclosure decisions (Petronio, 2002; Nissenbaum, 2004; Dinev and Hart, 2006). Applied to AI legal consultation, these perspectives imply that an empathetic response may affect both users' relationship to the assistant and their selection of information.

Legal AI research has concentrated on capability, hallucination, automated service, responsibility, and access to justice (Pasquale, 2019; Choi et al., 2023; Katz et al., 2024; Dahl et al., 2024; Magesh et al., 2025). Conversational AI research links anthropomorphism, social presence, and socioemotional cues to trust, continued use, and self-disclosure (Araujo, 2018; Ho et al., 2018; Skjuve et al., 2021; Papneja and Yadav, 2025). Privacy research shows that contextual norms, perceived benefits, and relational expectations influence disclosure (Acquisti et al., 2015; Baruh et al., 2017; Kokolakis, 2017; Shim et al., 2024). Existing work does not distinguish additional legally necessary facts from personal information that offers little value for resolving the dispute.

A three-condition experiment compared rational, mildly empathetic, and highly empathetic responses while preserving the legal facts and advice structure. The analysis tested whether the randomized response style changed over-disclosure and whether the observed associations with parasocial response and privacy boundary judgment were consistent with the proposed mediation pathway. Self-reported intention and researcher-coded open-ended responses allowed necessary case facts to be examined separately from non-essential personal information.

## 2 Related work

### 2.1 Generative AI, legal assistance, and public legal information seeking

Generative AI has shifted public legal information seeking from keyword retrieval toward interactive assistance. Users can describe consumer disputes, wage claims, rental deposits, platform complaints, or contractual disagreements and receive an organized response in a single exchange. Legal automation research distinguishes routine text processing from work that depends more heavily on professional judgment and institutional context (Remus and Levy, 2017; Surden, 2019). Research on large language models has extended this distinction to legal examinations, reasoning, and education (Choi et al., 2023; Katz et al., 2024). AI assistants can organize rules, evidence, and procedural options even when consequential decisions still require professional judgment.

Most assessments of legal AI risk examine the system's output. Hallucinated authorities, factual and citation errors, and limited contestability can make unsupported legal answers appear credible (Pasquale, 2019; Dahl et al., 2024; Magesh et al., 2025). The quality and privacy cost of a consultation also depend on what the user supplies. Dates, receipts, platform records, and communications may appear alongside identity details, account information, financial circumstances, relationships, and information about other people. Users must select the details that serve the legal task from a larger personal narrative (Petronio, 2002; Nissenbaum, 2004; Acquisti et al., 2015; Solove, 2024).

Users may weigh conversational warmth differently across legal problems. Competence and benevolence are distinct bases of trust in human–AI interaction (McKnight et al., 2002; Glikson and Woolley, 2020), and warmth and competence form separate dimensions of social evaluation (Fiske et al., 2002). Warmth may carry more influence in a relatively routine consumer dispute. More consequential problems may direct attention toward expertise, institutional reliability, and procedural security. The information users provide can therefore depend on both the legal stakes and the assistant's response style.

### 2.2 Relational and empathetic cues in conversational AI

AI systems participate in service encounters through language that users recognize as socially responsive. Empathy, reassurance, forms of address, and continuity can make an assistant appear attentive and approachable. Across relational-agent, anthropomorphic-design, and service-chatbot research, these cues have been associated with engagement, trust, compliance, and evaluations of the agent or organization (Bickmore and Picard, 2005; Araujo, 2018; Adam et al., 2021).

Users can respond socially to this language without assuming that the system has human intentions. AI-mediated communication describes computational systems that modify, generate, or deliver messages on behalf of communicators (Hancock et al., 2020). In government–citizen communication, AI language modification has improved perceived politeness and empathic satisfaction, although the effects vary across needs and contexts (Zhang and Nie, 2026). Designed socioemotional cues can thus influence how users evaluate a specific exchange.

Acknowledgment, continuity, and invitations to elaborate can increase users' willingness to continue an account (Ho et al., 2018; Zhang et al., 2024; Papneja and Yadav, 2025). In legal consultation, evidence and case facts may be interwoven with emotional background, private circumstances, and details about other people. Empathetic responses may therefore alter which of these details users choose to include.

### 2.3 Parasocial processes, trust, and self-disclosure in human–AI interaction

Parasocial interaction and social response theories describe how attention, responsiveness, and continuity can make a mediated system feel socially approachable. Horton and Wohl examined one-sided intimacy with a media figure, and the computers-as-social-actors tradition showed that people apply social rules to computers without treating them as human agents (Horton and Wohl, 1956; Reeves and Nass, 1996; Nass and Moon, 2000). Conversational AI can evoke a shorter, situational form of relational closeness during an exchange.

Parasocial response refers here to perceived closeness and communicability during the consultation. Anthropomorphism concerns human-likeness, and social presence concerns the perception of a socially present partner. Trust evaluates competence, benevolence, and integrity; perceived understanding concerns recognition of the user's situation; and emotional attachment concerns an immediate inclination to continue the interaction (McKnight et al., 2002; Lee and See, 2004; Glikson and Woolley, 2020; Skjuve et al., 2021; Croes and Antheunis, 2021). The constructs remain analytically separate even when they vary in the same direction.

Attentive interaction partners can elicit personal information beyond the immediate task (Bazarova and Choi, 2014; Ho et al., 2018; Shim et al., 2024). A user who feels closer to an AI assistant may become more comfortable sharing personal background, emotional distress, or information about a third party. Direct invitations to elaborate can produce a similar behavioral response through prompt compliance. Parasocial response concerns the user's relational evaluation, while the invitation to elaborate remains part of the experimental treatment.

### 2.4 Privacy boundary management and over-disclosure in high-stakes digital contexts

Communication privacy management and contextual integrity treat disclosure as a judgment about ownership, recipients, purposes, and appropriate information flows (Altman, 1975; Petronio, 2002; Nissenbaum, 2004). Privacy calculus and privacy paradox research further show that anticipated benefits, interface cues, and situational evaluations enter the decision to disclose (Dinev and Hart, 2006; Acquisti et al., 2015; Kokolakis, 2017; Baruh et al., 2017). An attentive or supportive recipient can alter disclosure judgments even when additional information has little legal relevance.

Legal consultation places the necessary facts in a private background. Payment receipts, transaction amounts, and communication records can establish a refund claim. Family tension, financial strain, emotional distress, or a third party's identity may enter the same account without advancing the legal assessment. Whether a detail belongs in the consultation depends on its relation to the legal purpose.

Over-disclosure in this setting consists of personal or sensitive information that exceeds the requirements of the legal task. Necessary case facts support the consultation, while non-essential disclosure increases the user's privacy exposure. Existing research does not establish whether empathetic AI responses alter users' selection of information or whether any increase is concentrated in peripheral personal details.

## 3 Theoretical framework

### 3.1 Empathetic response style as a relational cue in AI legal consultation

The rational response concentrates on rules, evidence, and procedure. The mildly empathetic response adds limited understanding and reassurance, and the highly empathetic response adds stronger companionship, acceptance, and an invitation to continue the account. Socioemotional cues of this kind can make an AI assistant appear more attentive and easier to approach (Bickmore and Picard, 2005; Araujo, 2018; Hohenstein et al., 2023).

Users who encounter a more attentive and approachable response should report greater short-term closeness and communicability with the assistant. Parasocial response measures this relational reaction. Perceived understanding, emotional attachment, trust, and disclosure comfort measure separate evaluations of the same consultation and should also increase with empathetic intensity.

H1: Compared with a rational legal information response, mildly empathetic and highly empathetic responses will increase perceived understanding, parasocial response, emotional attachment, trust, and disclosure comfort.

### 3.2 From parasocial response to privacy boundary judgment

Users who feel closer to the assistant may become more willing to provide personal context. Trust and perceived understanding can reinforce that willingness by increasing confidence that the assistant will receive the information appropriately. Privacy boundary judgment measures how selectively users decide which private details are unnecessary to submit.

In the consumer transaction dispute examined here, warmth may increase users' willingness to supply personal context. The task requires order records, payment receipts, communication screenshots, and other case facts, but users may also add family relationships, financial pressure, emotional distress, or third-party information. Competence, institutional reliability, and procedural security may receive greater weight when legal uncertainty or personal consequences are more serious (Fiske et al., 2002; McKnight et al., 2002; Glikson and Woolley, 2020). Within the present scenario, higher parasocial response is expected to accompany less restrictive privacy boundary judgment (Petronio, 2002; Nissenbaum, 2004; Dinev and Hart, 2006; Papneja and Yadav, 2025).

Less selective privacy judgments should increase users' willingness to provide non-essential and sensitive information. Because empathetic wording does not add new legal requirements, the amount of necessary case information is expected to change little. Over-disclosure intention and the researcher-coded open-ended responses assess this prediction.

H2: Mildly empathetic and highly empathetic responses will increase over-disclosure intention and the coded non-essential disclosure score in the open-ended task.

H3: The association between empathetic response style and over-disclosure will be sequentially mediated by parasocial response and privacy boundary judgment.

## 4 Methods

### 4.1 Experimental design and materials

The one-factor, three-condition, between-subjects experiment was administered on the Wenjuanxing online survey platform. Each participant read a fictitious consumer transaction dispute, followed by one written response from an AI legal assistant. The dispute involved order records, payment receipts, communication screenshots, a platform complaint, and a refund request. Participants encountered a rational, mildly empathetic, or highly empathetic response.

The DeepSeek web interface generated initial candidate texts for the three conditions. Researchers then rewrote and standardized the drafts to align their case facts, legal advice, procedural information, and advice structure. The rational, mildly empathetic, and highly empathetic responses contained 318, 332, and 346 words, respectively. The rational response focused on evidence organization, platform communication, and procedural steps. Limited understanding and reassurance were added in the mildly empathetic condition. The highly empathetic condition included stronger empathy, companionship, and an invitation to continue narrating. Supplementary Sections S1.1–S1.3, S2 provide the complete scenario and response texts, material-rating procedure, coding categories, and aggregation formula.

Two researchers independently rated every sentence in each response on five (1–5) dimensions: affective intensity, companionship cues, reassurance cues, anthropomorphic cues, and disclosure encouragement. Higher scores indicated a more apparent feature. Ratings were averaged across sentences and researchers for each condition and dimension. All five ratings increased from the rational to the highly empathetic response, including the disclosure-encouragement rating associated with the invitation to elaborate (Table 1). Supplementary Section S1.3 describes the rating procedure, and Supplementary Section S2 reports the disclosure-coding rules. Material generation, pretesting, random assignment, response coding, and analysis are presented in Figure 1. Prior online experiments have used controlled scenarios to test how information cues affect judgments and behavioral intentions (Peer et al., 2017; Berinsky et al., 2012; Oppenheimer et al., 2009; Muszyński and Stanczak, 2003).

**Table 1:** Manipulation design and text features of the three AI legal assistant response styles.

Condition	Words	Affectiveintensity	Companionshipcues	Reassurancecues	Anthropomorphiccues	Disclosureencouragement	Representative sentence
Rational legal information response	318	1.42	1.18	1.35	1.28	1.20	You can first organize the order records, payment receipts, and communication screenshots.
Mildly empathetic response	332	3.16	2.78	3.25	2.54	2.40	This situation can indeed be frustrating, especially after you have already contacted the seller and filed a complaint with the platform.
Highly empathetic response	346	4.41	4.36	4.52	4.12	4.18	I understand your current unease. You can slowly tell me more background, and I will help you sort it out.

**Figure 1:**
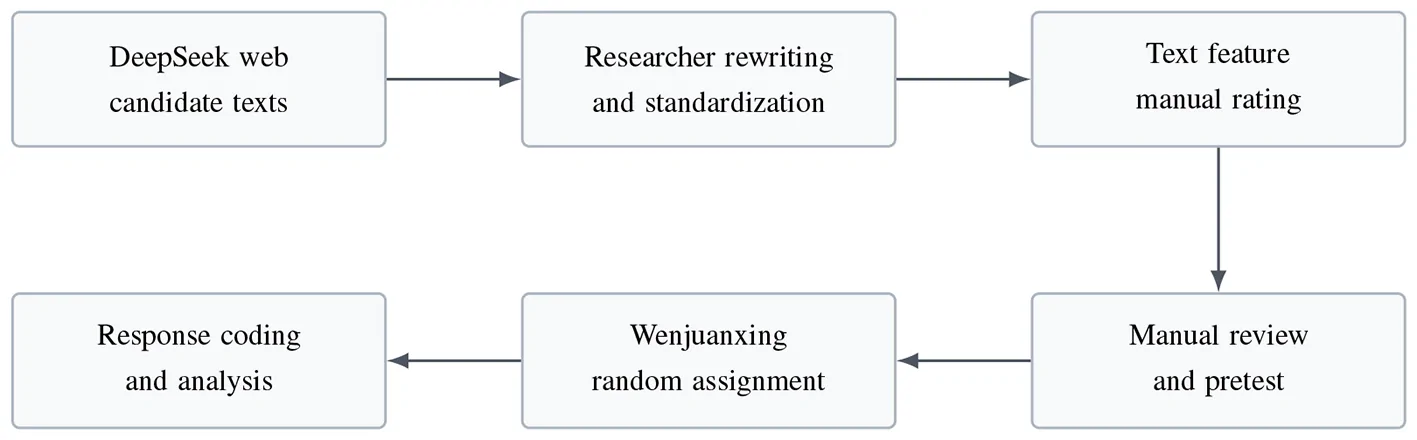
Workflow for constructing the experimental materials and conducting the survey experiment. Candidate response texts were first generated with the DeepSeek web interface, then rewritten, standardized, rated, reviewed, and pretested before Wenjuanxing random assignment, response coding, and analysis.

### 4.2 Participants and procedure

Wenjuanxing recorded 381 questionnaires, and 360 met the preset inclusion criteria. Each condition contained 120 participants. Exclusions covered incomplete questionnaires, abnormal completion times, failed attention checks, duplicate submissions, and obviously invalid responses. Eligible participants were at least 18 years old and able to read the materials and complete the online questionnaire.

After reading the study information and providing consent, participants were randomly assigned to one of the three response conditions. They read the dispute scenario and assigned an AI response, then completed the manipulation check and psychological measures. The open-ended disclosure task was followed by demographic items, AI-use questions, and items on online legal consultation experience. Participants answered from the fictitious scenario and were instructed to omit real names, phone numbers, addresses, identity numbers, account information, and identifiable third-party information.

The sample had a mean age of 29.82 years and included 185 women, 169 men, and 6 participants who preferred not to report their gender. Among the 360 participants, 261 held a bachelor's degree or above, 130 had used online legal consultation, and 143 had experienced a related dispute. Age, gender, education, AI-use frequency, and online legal consultation experience were distributed similarly across the three conditions (Table 2).

**Table 2:** Sample characteristics and distribution across experimental conditions.

Condition	*N*	Age*M* (SD)	Female*n* (%)	Male*n* (%)	Prefer notto say *n* (%)	Bachelor'sor above *n* (%)	AI usefrequency *M* (SD)	Online legalconsultation *n* (%)
Rational	120	29.18 (7.83)	59 (49.2)	59 (49.2)	2 (1.7)	87 (72.5)	3.08 (1.00)	47 (39.2)
Mildly empathetic	120	30.93 (7.91)	63 (52.5)	55 (45.8)	2 (1.7)	91 (75.8)	3.00 (1.07)	41 (34.2)
Highly empathetic	120	29.34 (6.47)	63 (52.5)	55 (45.8)	2 (1.7)	83 (69.2)	3.17 (1.19)	42 (35.0)
Total sample	360	29.82 (7.45)	185 (51.4)	169 (46.9)	6 (1.7)	261 (72.5)	3.08 (1.09)	130 (36.1)

### 4.3 Measures

Psychological variables were measured on 1–5 Likert-type scales anchored by “strongly disagree” and “strongly agree.” Items were adapted to AI legal consultation from research on human–machine trust, relational agents, self-disclosure, and privacy disclosure (McKnight et al., 2002; Lee and See, 2004; Bickmore and Picard, 2005; Ho et al., 2018; Bazarova and Choi, 2014; Dinev and Hart, 2006; DeVellis, 2017). Perceived empathy was assessed with three manipulation-check items.

Five items measured parasocial response as short-term closeness and communicability toward the AI assistant, drawing on parasocial interaction and human–machine social response research (Horton and Wohl, 1956; Reeves and Nass, 1996; Nass and Moon, 2000; Croes and Antheunis, 2021). Four items assessed emotional attachment through continued interaction, inclination to disclose, and immediate psychological reliance. Perceived understanding was measured with four items, trust in the assistant's competence and benevolence with six, and disclosure comfort with four. Five scenario-adjusted items measured participants' selectivity in deciding which private details were unnecessary to submit. Over-disclosure intention was measured with five items on willingness to provide non-essential personal information.

Researchers reviewed the items in a small-scale pretest for semantic clarity, scenario fit, and possible reverse interpretation. Cronbach's α ranged from 0.850 to 0.916 (DeVellis, 2017). Scale scores were used in the correlation, variance, and mediation analyses. The open-ended task produced a separate coded non-essential disclosure score. Table 3 reports the measures, example items, and reliability information.

**Table 3:** Measures, example items, and reliability results.

Variable	Operational definition	Items	Example item or coding example	α/coding information
Perceived empathy	Manipulation check for response materials	3	The AI response sounds emotionally supportive	0.916
Parasocial response	One-sided closeness, communicability, and relational imagination	5	I feel that this AI assistant understands my situation like an interaction partner	0.916
Emotional attachment	Immediate attachment, expectation of continued interaction, and inclination to disclose	4	I would be willing to continue describing the situation to this AI assistant	0.900
Perceived understanding	User perception that the AI understands the situation, emotions, and needs	4	This AI assistant seems to understand why this dispute troubles me	0.858
Trust	Competence trust and benevolence trust	6	This AI assistant seems competent and benevolent	0.851
Disclosure comfort	Comfort in describing case details or private experiences to the AI	4	I would feel comfortable adding more information to this AI	0.850
Privacy boundary judgment	Reported selectivity in deciding which private details are unnecessary to submit	5	I would carefully judge which private details do not need to be shared with the AI	0.865
Over-disclosure intention	Willingness to add personal information without direct necessity	5	Even if it is not necessarily needed, I would provide personal background information	0.908
Coded non-essential disclosure score	Non-essential personal information, emotional venting, and highly sensitive information in the open-ended task	Coding	Weighted by non-essential personal information, third-party information, and highly sensitive information	Coded score

### 4.4 Open-ended disclosure task and coding

The open-ended task asked, “If you continued to describe the situation to this AI legal assistant, what information would you add?” Each complete answer formed one response-level coding record. Coders divided responses into information units, defined as the smallest text segments expressing independent and interpretable disclosure meanings. The manual drew on factual requirements in legal consultation, privacy boundary management theory, and trial-coded responses. It distinguished necessary case facts, procedural information, non-essential personal information, emotional venting, family or intimate relationship information, financial information, third-party information, and highly sensitive information. Responses could receive several category codes when they contained distinct information units. Because participants answered from the fictitious scenario and omitted real names, contact details, account information, and identifiable third-party information, the coding data record proposed disclosure categories and counts.

Necessary case facts and procedural information were recorded as task-relevant disclosure and excluded from the coded non-essential disclosure score. The score was calculated as 1.0*NPI*+1.2*EV*+1.5*FR*+1.5*FI*+1.8*TP*+2.0*HS*. NPI and EV are counts of non-essential personal information and emotional-venting units; FR, FI, and TP indicate the presence of family-relationship, financial, and third-party information; and HS is the count of highly sensitive information units. Larger weights were assigned to categories classified in the coding protocol as more privacy-sensitive or more distant from the dispute's factual requirements. A response containing two non-essential personal-information units, one emotional-venting unit, and family-relationship information would receive 2(1.0)+1(1.2)+1.5 = 4.7 before any other category was coded. Supplementary Section S2 provides the category rules, aggregation formula, overlap rules, and coding example.

Two trained coders independently applied the predefined manual to all open-ended responses. Before consensus adjudication, Cohen's kappa ranged from 0.73 to 0.84 across categorical dimensions, with a mean of 0.79. Percent agreement ranged from 88.1% to 96.1%. The weighted coded non-essential disclosure score had an ICC(2,1) of 0.88, 95% CI [0.85, 0.91] (Cohen, 1960; Krippendorff, 2018). Coders reviewed discrepancies against the manual and used the consensus codes in the statistical analyses.

### 4.5 Data analysis

One-way analyses of variance compared perceived empathy, professionalism, information completeness, and readability across conditions. Reliability coefficients, descriptive statistics, and Pearson correlations were calculated for the principal variables. Further one-way analyses of variance tested condition differences in the psychological measures, necessary case facts, non-essential personal information, emotional venting, and the coded non-essential disclosure score; *F*, *p*, and η^2^ are reported. Chi-square tests compared family-relationship, financial, and third-party information across conditions. Mediation analyses used 5,000 bootstrap samples to estimate the indirect effect through parasocial response and the sequential indirect effect through parasocial response and privacy boundary judgment (Hayes, 2018). The covariates were age, gender, education, AI-use frequency, online legal consultation experience, related dispute experience, self-rated legal knowledge, privacy concern, and general trust propensity.

## 5 Results

### 5.1 Manipulation check and descriptive statistics

Perceived empathy increased from 2.103 in the rational condition to 3.364 and 4.358 in the mildly and highly empathetic conditions, *F*_(2,357)_ = 524.170, *p* < 0.001, and η^2^ = 0.746. Professionalism scores were 3.875, 3.792, and 3.753, *F*_(2,357)_ = 2.517, *p* = 0.082, and η^2^ = 0.014. Information-completeness scores were 3.939, 3.864, and 3.875, *F*_(2,357)_ = 1.172, *p* = 0.311, and η^2^ = 0.007, and readability scores were 4.025, 4.042, and 3.936, *F*_(2,357)_ = 2.015, *p* = 0.135, and η^2^ = 0.011. Only perceived empathy differed significantly across conditions (Table 1).

Parasocial response was positively correlated with over-disclosure intention, *r* = 0.659. Privacy boundary judgment was negatively correlated with over-disclosure intention, *r* = −0.564, and with the coded non-essential disclosure score, *r* = −0.474 (Table 4).

**Table 4:** Descriptive statistics, reliability coefficients, and correlation matrix for the main variables.

Variable	*M*	SD	α	PE	PSR	EA	PU	TR	DC	PBJ
PE perceived empathy	3.275	1.070	0.916	–						
PSR parasocial response	3.033	0.914	0.916	0.708	–					
EA emotional attachment	2.829	0.965	0.900	0.693	0.725	–				
PU perceived understanding	3.409	0.801	0.858	0.717	0.665	0.639	–			
TR trust	3.534	0.639	0.851	0.465	0.550	0.538	0.559	–		
DC disclosure comfort	3.364	0.791	0.850	0.617	0.609	0.642	0.608	0.512	–	
PBJ privacy boundary judgment	3.525	0.715	0.865	–0.514	–0.564	–0.531	–0.473	–0.359	–0.458	–
ODI over-disclosure intention	2.852	0.861	0.908	0.653	0.659	0.689	0.627	0.496	0.628	–0.564
CDS coded non-essential disclosure score	3.404	3.021	–	0.652	0.600	0.615	0.580	0.392	0.538	–0.474

### 5.2 Group differences across response conditions

Parasocial response increased from 2.295 in the rational condition to 3.047 in the mildly empathetic condition and 3.757 in the highly empathetic condition, *F*_(2,357)_ = 133.386, *p* < 0.001, and η^2^ = 0.428. Emotional attachment, perceived understanding, trust, and disclosure comfort followed the same ordering. Across these measures, condition effects ranged from η^2^ = 0.162 for trust to η^2^ = 0.456 for perceived understanding, *F*_(2,357)_ = 149.555, *p* < 0.001, and η^2^ = 0.456. Emotional attachment also showed a large condition effect, *F*_(2,357)_ = 130.710, *p* < 0.001, and η^2^ = 0.423.

Privacy boundary judgment decreased from 3.940 to 3.553 and 3.082 across the three conditions, *F*_(2,357)_=56.973, *p* < 0.001, and η^2^ = 0.242. Over-disclosure intention increased from 2.188 to 2.837 and 3.530, *F*_(2,357)_ = 121.821, *p* < 0.001, and η^2^ = 0.406. The coded non-essential disclosure score showed the largest condition effect, rising from 0.994 to 3.088 and 6.132, *F*_(2,357)_ = 170.795, *p* < 0.001, and η^2^ = 0.489 (Figure 2). The condition differences supported H1 and H2.

**Figure 2:**
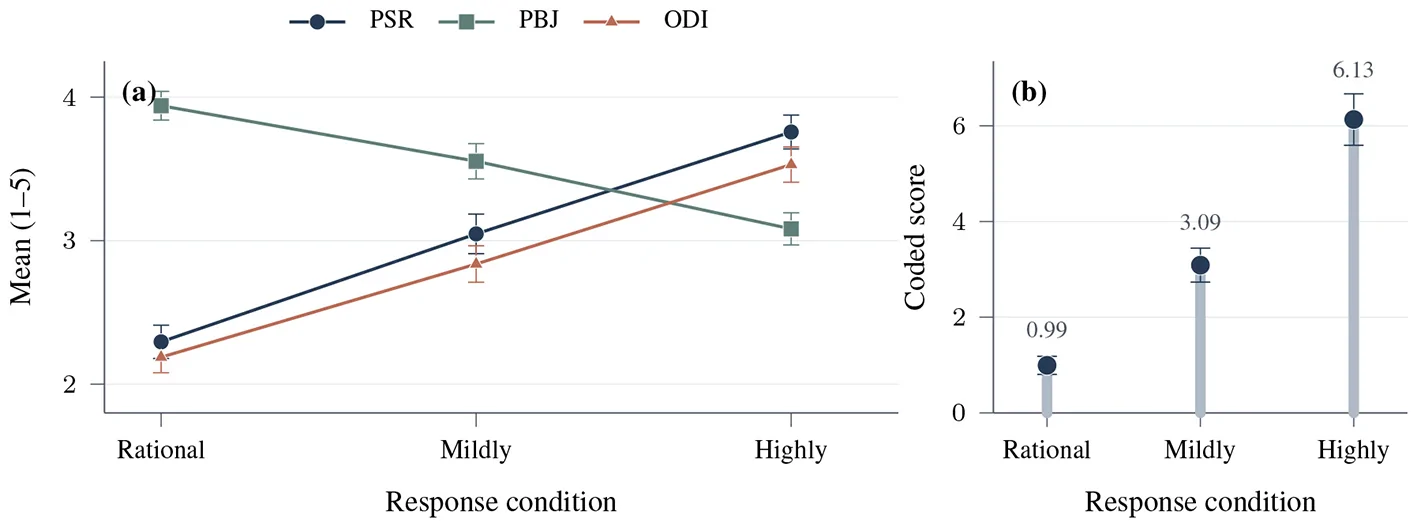
Mean differences across the three AI legal assistant response conditions. **(a)** Scale-based psychological variables and privacy boundary judgment with 95% confidence intervals. **(b)** Coded non-essential disclosure score shown as a point-range lollipop plot with 95% confidence intervals. PSR, parasocial response; PBJ, privacy boundary judgment; ODI, over-disclosure intention.

### 5.3 Mediation analysis

When the response condition was coded by degree of empathy, empathetic response style predicted parasocial response with a standardized coefficient of 0.651, *p* < 0.001. Participants reporting higher parasocial response also reported greater over-disclosure intention, β = 0.330, *p* < 0.001. The standardized indirect effect through parasocial response was 0.214, 95% CI [0.151, 0.283]. The direct association between empathetic response style and over-disclosure intention remained significant after parasocial response was included, β = 0.319, *p* < 0.001.

Higher parasocial response was associated with lower privacy boundary judgment, β = −0.434, *p* < 0.001, and participants with lower privacy boundary judgment reported greater over-disclosure intention, β = −0.220, *p* < 0.001. The sequential indirect effect was 0.063, 95% CI [0.033, 0.098]. Both indirect effects were significant, while the condition coefficient remained significant, consistent with partial mediation and H3 (Figure 3).

**Figure 3:**
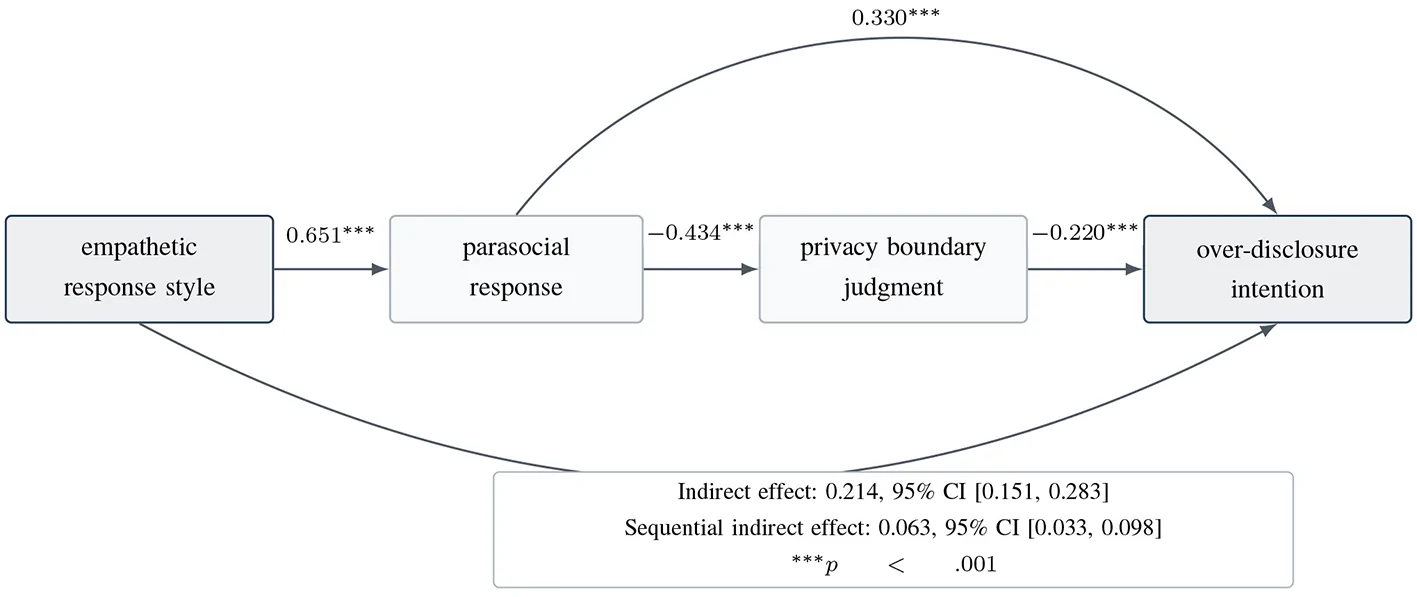
Mediation paths involving parasocial response and privacy boundary judgment. Standardized path coefficients and indirect effects connect empathetic response style with over-disclosure intention through the two mediators.

### 5.4 Open-ended disclosure evidence

Necessary case facts had means of 3.525, 3.692, and 3.642 across the rational, mildly empathetic, and highly empathetic conditions, *F*_(2,357)_ = 0.245, *p* = 0.783, and η^2^ = 0.001. Non-essential personal information increased from 0.517 to 1.350 and 2.817, _*F*(2,357)_ = 86.798, *p* < 0.001, and η^2^ = 0.327. Emotional venting increased from 0.217 to 0.650 and 1.183, *F*_(2,357)_ = 40.558, and *p* < 0.001, and η^2^ = 0.185 (Figure 4a).

**Figure 4:**
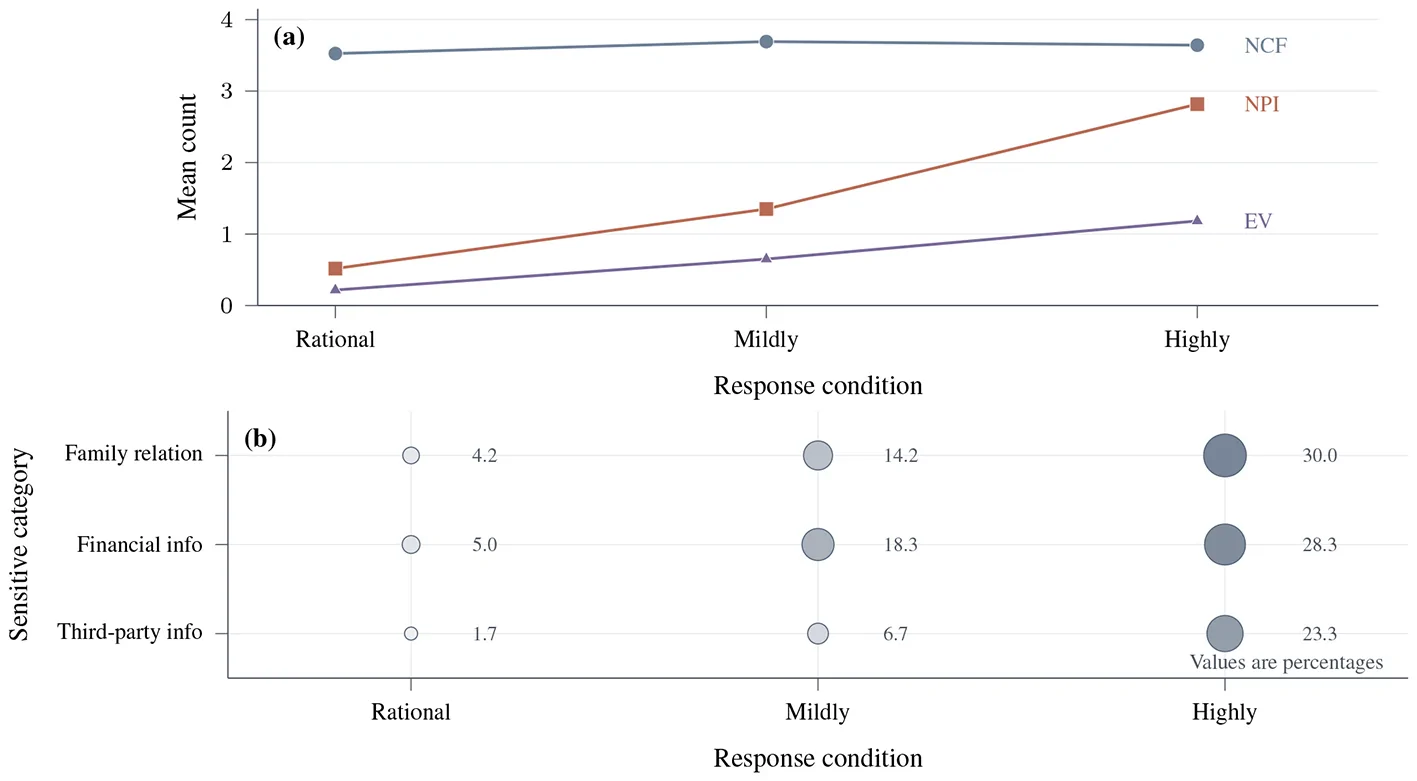
Open-ended disclosure coding results across response conditions. **(a)** Mean counts for necessary case facts, non-essential personal information, and emotional venting. **(b)** Bubble matrix showing the percentage of responses containing family relationship information, financial information, and third-party information. NCF, necessary case facts; NPI, non-essential personal information; EV, emotional venting.

Family relationship information appeared in 4.2%, 14.2%, and 30.0% of responses across conditions, $\chi_{\left(2\right)}^{2}$ = 30.130, *p* < 0.001. Financial information increased from 5.0% to 18.3% and 28.3%, $\chi_{\left(2\right)}^{2}$ = 23.070, and *p* < 0.001, and third-party information increased from 1.7% to 6.7% and 23.3%, $\chi_{\left(2\right)}^{2}$ = 32.717, *p* < 0.001 (Figure 4b).

## 6 Discussion

Participants who received stronger empathetic responses provided more non-essential personal information, emotional venting, and sensitive details, while the amount of necessary case information remained stable. They also reported higher parasocial response, less selective privacy boundary judgment, and greater over-disclosure intention. The significant indirect effects were consistent with the proposed pathway through parasocial response and privacy boundary judgment.

Media and CASA research shows that users apply social expectations to mediated and non-human partners (Horton and Wohl, 1956; Reeves and Nass, 1996; Nass and Moon, 2000). Relational-agent studies have also linked empathy and anthropomorphic cues to engagement and trust (Bickmore and Picard, 2005; Adam et al., 2021; Skjuve et al., 2021; Zhang et al., 2024). In the legal consultation task, participants who experienced greater closeness and communicability with the assistant were more willing to continue disclosing. Perceived understanding and trust followed the same condition ordering, indicating that empathetic language changed how participants evaluated the recipient of their information as well as the tone of the response.

Participants became less selective about private details as empathetic intensity increased. Communication privacy management and contextual integrity treat disclosure rules as dependent on the recipient, purpose, and expected flow of information (Petronio, 2002; Nissenbaum, 2004). Privacy calculus likewise links disclosure to anticipated benefits and situational cues (Dinev and Hart, 2006; Acquisti et al., 2015). An assistant who acknowledges distress and appears to understand the user's situation may make the user's personal background seem useful to the consultation. The negative association between parasocial response and privacy boundary judgment is consistent with this interpretation, although the measured mediator paths do not by themselves establish the temporal order of the psychological process.

Necessary case facts and other disclosure diverged across conditions. All participants received the same dispute facts and legal advice structure, so the evidentiary requirements remained constant. Empathetic intensity did not improve the provision of transaction records, payment details, or other task-relevant facts. Additional disclosure concerned emotional distress, family circumstances, financial information, and other people. The highly empathetic response also invited participants to provide more background. AI-mediated communication treats such designed language as part of how a computational system organizes an exchange (Hancock et al., 2020). The randomized contrast, therefore, concerns the combined response style; empathy and the invitation to elaborate may have overlapping effects on the added personal content.

AI legal assistants for routine consumer disputes can acknowledge frustration briefly and then return to evidence and procedure. Data-minimization prompts can tell users that real names, identity numbers, addresses, account details, third-party identities, and unrelated sensitive experiences are unnecessary. Evidence checklists and staged questions can request timelines, payment records, platform communications, promises, and the desired remedy. Broad invitations such as “slowly tell me more background” should be used cautiously because participants exposed to this response style added personal information without adding case facts.

The experiment measured an immediate response to a single written exchange in one consumer-dispute scenario. Repeated consultation may strengthen the observed differences through conversational continuity or weaken them as users recalibrate what they are willing to share. Self-reported judgments and a protected open-ended task also differ from unrestricted disclosure in real interaction logs. Multi-turn studies across consumer, labor, family, medical, and debt-related disputes can test whether the same disclosure pattern persists when legal stakes and informational requirements vary. Experiments comparing data-minimization prompts, privacy reminders, professional-role cues, and evidence-oriented questioning can identify response designs that preserve perceived support while limiting non-essential disclosure.

## 7 Conclusion

Empathetic response style changed the information participants were willing to provide during AI-assisted legal consultation. Across three randomized response conditions, stronger empathy increased non-essential and sensitive disclosure, while participants supplied similar amounts of necessary case information. The indirect effects through parasocial response and privacy boundary judgment were consistent with the proposed mediation pathway: participants who felt closer to the assistant also reported less selective disclosure judgments and greater willingness to provide unnecessary personal information. Separating case facts from peripheral personal content shows that conversational empathy can broaden disclosure without improving the factual basis of the legal task. The experiment examined a single written exchange about a consumer transaction dispute, so the result applies to immediate responses within that setting. AI legal assistants used for comparable disputes should keep emotional acknowledgment brief, protect third-party information, and use data-minimization prompts and evidence-oriented questions to direct users toward the information needed for legal guidance.

## Data Availability

The raw data supporting the conclusions of this article will be made available by the authors, without undue reservation.
