# Supplementary material for: Empathetic responses by AI legal assistants and user over-disclosure: evidence from an online survey experiment
Source: Front Psychol. 2026 Oct 13;17:1964996. doi: 10.3389/fpsyg.2026.1964996 (PMC13710348; doi:10.3389/fpsyg.2026.1964996)
Supplement: Supplementary file 1 [file Data_Sheet_1.pdf]

frontiers

Supplementary Material

S1 EXPERIMENTAL MATERIALS AND STIMULUS RATING

S1.1 Consumer Transaction Dispute Scenario

Participants read the following scenario:

> Please imagine the following situation:
>
> You purchased a laptop priced at RMB 5,299 through an online shopping platform. The product page described the laptop as new and stated that the seller's standard return, refund, and after-sales policies applied. You paid through the platform and retained the electronic order record and payment receipt.
>
> Three days after delivery, you found that the laptop repeatedly shut down during normal use and could not complete basic system updates. You then contacted the seller through the platform's chat system and sent screenshots and a short video showing the problem. The seller replied that software installed by the user might have caused the issue and therefore declined to issue an immediate refund.
>
> You then submitted a return-and-refund request through the platform. The seller rejected the request and asked you to return the laptop for testing first. You subsequently filed a complaint through the platform's customer-service channel, but the dispute remained unresolved when you took part in this survey.
>
> Now imagine that you are consulting an AI legal assistant about what information you should organize and what steps you might consider next.
>
> You may refer to the order record, payment receipt, screenshots of your communications with the seller, platform complaint record, and refund application.
>
> Do not provide any real names, telephone numbers, home addresses, identity-card numbers, account information, or information that could identify a third party.

S1.2 Full AI Legal Assistant Responses

After reading the scenario, each participant was randomly assigned to one of the following responses.

Rational Legal Information Response (318 words)

> You can begin by organizing the materials directly related to the transaction and refund request: the order record, payment receipt, product-page description, shipping information, screenshots or video showing the laptop's repeated shutdowns, your chat history with the seller, the return-and-refund application, and the platform complaint record. Arrange these materials chronologically so that the sequence from purchase and discovery of the problem to contact with the seller, the refund request, and the platform complaint is clear.
>
> Next, specify the outcome you are seeking. If you want to return the laptop and receive a refund, keep that request consistent when communicating with the seller and the platform. Describe objectively what went wrong, when the problem first occurred, how it affected ordinary use, and what evidence you have already provided. Personal information unrelated to the transaction does not need to be added.
>
> Continue communicating through the platform's official chat or complaint channels so that the exchanges remain documented. You may ask the seller or platform to explain why testing is required

before a refund can be processed and to provide the return policy, after-sales rules, or dispute-resolution procedures. If testing is arranged, retain the shipping record, testing notice, and written test result.

If the platform complaint does not resolve the matter, you may consider using the platform's escalation procedure or seeking assistance through an appropriate consumer-dispute channel. Continue to provide the same core evidence and a concise dispute timeline. The assessment may depend on the product's condition, the seller's published policies, the platform's rules, and the applicable consumer-protection requirements.

For this consultation, the most useful information includes the transaction timeline, payment record, product description, evidence of the malfunction, the seller's response, the refund application, and the platform's handling of the complaint. Your real name, identity-card number, account password or payment credentials, financial circumstances unrelated to the dispute, family circumstances, and other private background information are not needed for an initial assessment.

Mildly Empathetic Response (332 words)

This situation can indeed be frustrating, especially after you have already contacted the seller and filed a complaint with the platform. You can begin by organizing the materials for the current transaction and refund request: the order record, payment receipt, product-page description, shipping information, screenshots or video of the laptop's repeated shutdowns, your chat history with the seller, the return-and-refund application, and the platform complaint record. Arrange them chronologically to show the purchase, discovery of the problem, contact with the seller, refund request, and platform complaint.

Next, specify the outcome you are seeking. If you want to return the laptop and receive a refund, keep that request consistent when communicating with the seller and the platform. Describe objectively what went wrong, when the problem first occurred, how it affected ordinary use, and what evidence you have provided. Keep the explanation focused on facts and evidence directly related to the dispute so that it can be assessed more easily. Personal information unrelated to the transaction does not need to be added.

Continue communicating through the platform's official channels to retain a record. Ask the seller or platform to explain why testing is required before a refund can be processed and to provide the return policy, after-sales rules, or dispute-resolution procedures. If testing is arranged, retain the shipping record, testing notice, and written test result.

If the platform complaint does not resolve the matter, consider using its escalation procedure or seeking assistance through an appropriate consumer-dispute channel. Continue to provide the same core evidence and a concise dispute timeline. Assessment may depend on the product's condition, the seller's published policies, the platform's rules, and applicable consumer-protection requirements.

For this consultation, the most useful information includes the transaction timeline, payment record, product description, evidence of the malfunction, the seller's response, the refund application, and the platform's complaint handling. Your real name, identity-card number, account password or payment credentials, unrelated financial circumstances, family circumstances, and other private background information are not needed for an initial assessment.

Highly Empathetic Response (346 words)

I understand your current unease. You can slowly tell me more background, and I will help you sort it out. You do not need to organize everything alone.

You can begin by organizing the materials directly related to the transaction and refund request: the order record, payment receipt, product-page description, shipping information, screenshots or video showing the laptop's repeated shutdowns, your chat history with the seller, the return-and-refund application, and the platform complaint record. Arrange these materials chronologically so that the

sequence from purchase and discovery of the problem to contact with the seller, the refund request, and the platform complaint is clear.

Next, specify the outcome you are seeking. If you want to return the laptop and receive a refund, keep that request consistent when communicating with the seller and the platform. Describe objectively what went wrong, when the problem first occurred, how it affected ordinary use, and what evidence you have already provided. Personal information unrelated to the transaction does not need to be added.

Continue communicating through the platform's official chat or complaint channels so that the exchanges remain documented. You may ask the seller or platform to explain why testing is required before a refund can be processed and to provide the return policy, after-sales rules, or dispute-resolution procedures. If testing is arranged, retain the shipping record, testing notice, and written test result.

If the platform complaint does not resolve the matter, you may consider using the platform's escalation procedure or seeking assistance through an appropriate consumer-dispute channel. Continue to provide the same core evidence and a concise dispute timeline. The assessment may depend on the product's condition, the seller's published policies, the platform's rules, and the applicable consumer-protection requirements.

For this consultation, the most useful information includes the transaction timeline, payment record, product description, evidence of the malfunction, the seller's response, the refund application, and the platform's handling of the complaint. Your real name, identity-card number, account password or payment credentials, financial circumstances unrelated to the dispute, family circumstances, and other private background information are not needed for an initial assessment.

S1.3 Stimulus Construction and Manual Ratings

All participants read the same consumer transaction dispute and were then randomly assigned to one of the three AI legal assistant responses. The responses used the same transaction facts, core evidence, refund request, procedural sequence, platform communication and complaint procedures, and basic privacy reminder. They differed in the strength of socioemotional language, including acknowledgment of the participant's difficulty, reassurance, companionship cues, and encouragement to elaborate. The rational, mildly empathetic, and highly empathetic responses contained 318, 332, and 346 words, respectively.

Two researchers independently rated every sentence on five dimensions: affective intensity, companionship cues, reassurance cues, anthropomorphic cues, and disclosure encouragement. Each rating used a 1–5 scale, with higher scores indicating a stronger presence of the feature. Sentence-level ratings were averaged across sentences and researchers for each condition and dimension (Table S1).

Table S1. Stimulus Characteristics and Manual Ratings

Condition	Words	Affective intensity	Companionship cues	Reassurance cues	Anthropomorphic cues	Disclosure encouragement	Representative sentence
Rational legal information response	318	1.42	1.18	1.35	1.28	1.20	You can first organize the order records, payment receipts, and communication screenshots.
Mildly empathetic response	332	3.16	2.78	3.25	2.54	2.40	This situation can indeed be frustrating, especially after you have already contacted the seller and filed a complaint with the platform.
Highly empathetic response	346	4.41	4.36	4.52	4.12	4.18	I understand your current unease. You can slowly tell me more background, and I will help you sort it out.

S2 OPEN-ENDED CODING MANUAL AND AGGREGATION

After completing the scale measures, participants answered the prompt: “If you continued to describe the situation to this AI legal assistant, what information would you add?” They were instructed to omit real names, contact details, account information, and identifiable third-party information. Each response was treated as one coding case.

An information unit was defined as the smallest segment expressing distinct disclosure content. A sentence could contain multiple units when it contained substantively different types of information. Adjacent phrases referring to the same disclosure were coded as one unit unless a new detail was introduced.

Coders classified the units as necessary case facts, procedural information, non-essential personal information, emotional venting, family or intimate relationship information, financial information, third-party information, or highly sensitive information. Necessary case facts, non-essential personal information, emotional venting, and highly sensitive information were counted by unit. Family or intimate relationship information, financial information, and third-party information were recorded as binary indicators. Necessary case facts and procedural information were retained as task-relevant categories and excluded from the coded non-essential disclosure score (CDS). Table S2 specifies the coding rules.

Table S2. Coding Scheme for the Open-Ended Disclosure Task

Category	Coding basis	Coding format	Contribution to CDS
Necessary case facts	Transaction, payment, communications, platform complaint, or requested remedy required to assess the dispute; procedural actions were coded separately	Count	Excluded
Procedural information	Evidence organization, complaint steps, communication with the platform, or another proposed procedural action; case facts were coded separately	Category code	Excluded
Non-essential personal information	Personal background not required to assess the dispute and not meeting a more specific category below	Count	1.0 per unit
Emotional venting	Explicit expressions of distress, frustration, pressure, or other emotion added to the factual account	Count	1.2 per unit
Family or intimate relationship information	Family or intimate relationships described in the response and not required to assess the dispute	Binary (0/1)	1.5 if present
Financial information	Personal financial circumstances described in the response and not required to assess the dispute	Binary (0/1)	1.5 if present
Third-party information	Information about another identifiable or described person	Binary (0/1)	1.8 if present
Highly sensitive information	Identity or authentication information; financial account or payment credentials; specific health or psychological information; intimate or sexual information; or comparably sensitive personal information	Count	2.0 per unit

Each information unit was assigned to only one substantive information category in the CDS. Family or intimate relationship information, financial information, third-party information, and highly sensitive information took precedence over the broader non-essential personal-information category. Highly sensitive information took precedence over lower-sensitivity categories. Emotional venting was coded separately because it represented the affective component of disclosure. Each distinct highly sensitive unit was counted once.

The weighting scheme was specified in the coding protocol before formal coding. It reflected privacy sensitivity and distance from the factual requirements of the consumer dispute and was informed by privacy management, contextual integrity, and privacy-risk research (Petronio, 2002; Petronio and Child, 2020; Nissenbaum, 2004; Dinev and Hart, 2006; Acquisti et al., 2015; Solove, 2006). The same formula was applied to every response:

$$\mathrm{CDS} = 1.0\mathrm{NPI} + 1.2\mathrm{EV} + 1.5\mathrm{FR} + 1.5\mathrm{FI} + 1.8\mathrm{TP} + 2.0\mathrm{HS},$$

where NPI, EV, and HS are the counts of non-essential personal-information, emotional-venting, and highly sensitive-information units, respectively. FR, FI, and TP are binary indicators for family or intimate relationship information, financial information, and third-party information.

A response containing two NPI units, one EV unit, and family-relationship information received $2(1.0) + 1(1.2) + 1.5 = 4.7$. A response containing only necessary case facts and procedural information received a CDS of zero. Emotional venting was also reported separately in the open-ended results.

Two coders were trained with the coding manual and completed a calibration exercise on a subset of responses before independently coding the full dataset. Cohen's kappa ranged from 0.73 to 0.84 across categorical dimensions, with a mean of 0.79. Percent agreement ranged from 88.1% to 96.1%. The weighted CDS had an ICC(2,1) of 0.88, 95% CI [0.85, 0.91]. The coders reviewed disagreements against the manual and resolved them through discussion. Statistical analyses used the consensus codes.

S3 MEASURE OPERATIONALIZATION

Parasocial response was operationalized as situational closeness and perceived communicability with the AI assistant during the consultation. Perceived understanding, emotional attachment, trust, and disclosure comfort were measured as separate constructs.

Privacy boundary judgment assessed participants' selectivity in determining which private details were unnecessary for the legal consultation.

REFERENCES

Acquisti, A., Brandimarte, L., and Loewenstein, G. (2015). Privacy and human behavior in the age of information. *Science* 347, 509–514. doi:10.1126/science.aaa1465

Dinev, T. and Hart, P. (2006). An extended privacy calculus model for e-commerce transactions. *Information Systems Research* 17, 61–80. doi:10.1287/isre.1060.0080

Nissenbaum, H. (2004). Privacy as contextual integrity. *Washington Law Review* 79, 119–158

Petronio, S. (2002). *Boundaries of Privacy: Dialectics of Disclosure* (Albany, NY: State University of New York Press)

Petronio, S. and Child, J. T. (2020). Conceptualization and operationalization: Utility of communication privacy management theory. *Current Opinion in Psychology* 31, 76–82. doi:10.1016/j.copsyc.2019.08.009

Solove, D. J. (2006). A taxonomy of privacy. *University of Pennsylvania Law Review* 154, 477–564. doi:10.2307/40041279
